# Awareness of Virus–Cancer Links and Willingness to Vaccinate Against a Cancer-Associated Virus by HPV Vaccination Status Among Polish Students: A Cross-Sectional Study

**DOI:** 10.3390/vaccines14040335

**Published:** 2026-04-09

**Authors:** Anita Mikołajczyk, Emilia Lemkowska, Mateusz Mikołajczyk

**Affiliations:** 1 Department of Psychology and Sociology of Health and Public Health, Collegium Medicum, University of Warmia and Mazury in Olsztyn, Warszawska Str. 30, 10-082 Olsztyn, Poland; 2 Student Scientific Group of Public Health Research, Collegium Medicum, University of Warmia and Mazury in Olsztyn, Warszawska Str. 30, 10-082 Olsztyn, Poland; elemkowska123@gmail.com; 3 Masovian Dental Center Ltd., Nowy Zjazd Str. 1, 00-301 Warsaw, Poland; matmateusza@gmail.com

**Keywords:** human papillomavirus (HPV), virus–cancer awareness, HPV vaccination status, perceived infection risk, cancer prevention

## Abstract

Background/Objectives: Prevention of virus-related cancers is a multifaceted process shaped by vaccination and public awareness. This study assessed awareness of virus–cancer relationships and willingness to vaccinate against a cancer-associated virus among medical and non-medical students. We also evaluated whether human papillomavirus (HPV)-vaccinated students demonstrate greater awareness of the HPV-cancer link compared to unvaccinated students, and examined willingness to vaccinate against a certain cancer-associated virus according to HPV vaccination status. Methods: This cross-sectional survey was conducted in Poland (October 2023–June 2024) and included 1013 first- and second-year university students recruited via convenience sampling. Participation was voluntary and anonymous. Results: Awareness of virus–cancer relationships was low, ranging from 19% for Epstein–Barr virus-related cancers to 43.8% for HPV-related cervical cancer. Women were more likely than men to recognize the HPV–cervical cancer link (OR = 2.08, *p* < 0.001), supporting gender differences and the need for gender-neutral HPV education with targeted strategies for men. Medical students demonstrated higher awareness than non-medical students. HPV vaccination coverage was low (14.5%), with higher uptake among medical students (21.2% vs. 8.2%). Notably, 41.3% of non-medical students and 7.5% of medical students had never heard of HPV vaccination. Willingness to vaccinate against a cancer-associated virus varied according to perceived infection risk. Conclusions: These findings highlight the need for targeted educational interventions to improve awareness of HPV–cancer links and risk perception, as well as to ensure ongoing education of both HPV-vaccinated and unvaccinated individuals to support informed health decisions and vaccine acceptance.

## 1. Introduction

Cancer is one of the greatest challenges of modern medicine, being the second most common cause of death in the world after cardiovascular diseases [1,2,3]. In recent decades, research on cancer pathogenesis has revealed that certain viruses play a critical role in initiating neoplastic processes. In 2018, infectious agents, mainly bacterial and viral, classified by the International Agency for Research on Cancer (IARC) as Group 1 carcinogens, were responsible for 2.2 million new cancer cases, accounting for 13% of all cases [4]. Group 1 carcinogenic viruses to humans include hepatitis B virus (HBV), hepatitis C virus (HCV), Epstein–Barr virus (EBV), human papillomavirus (HPV), human T-cell lymphotropic virus type 1 (HTLV-1), Kaposi sarcoma-associated herpesvirus (KSHV), and human immunodeficiency virus (HIV), which can induce cancer through various mechanisms [5,6]. Recently, hepatitis D virus (HDV) and Merkel cell polyomavirus (MCPyV) were also classified as Group 1 carcinogens [7]. At the same time, human cytomegalovirus HCMV was classified as possibly carcinogenic to humans (Group 2B). The evidence from previous studies suggesting that HCMV exhibits the key characteristics of carcinogens was limited and insufficient, so further research is needed before it could be assigned to Group 1 [7,8,9]. Overall, the role of both oncoviruses and putative oncogenic viruses (viruses potentially associated with cancer development) is complex and multifaceted. Research into newly identified viruses potentially associated with cancer development needs scientific evidence with critical analysis [10].

Cancers associated with oncoviruses typically develop years or even decades after infection, as their onset is linked to long-term viral persistence. The state in which a complete copy of the viral genetic material remains in the infected host cell, but continuous production of infectious viral particles does not occur, is called latency. Immunosuppression can facilitate the reactivation of latent viral genomes, whereas host immunity may suppress the productive stages of the viral life cycle without completely eliminating viral genomes from infected basal cells. In these cells, viral antigen expression remains below detectable levels, thereby complicating complete eradication of the virus from the host. An alternative strategy, commonly employed by viruses such as HPV and EBV, is the establishment of chronic infection without lysis of the host cell. In this way, viruses can persist within the host for extended periods without immune detection, as viral gene expression is maintained at very low levels. Genome amplification occurs following cellular differentiation, and chronic viral infections can be reactivated under favorable conditions. During the latent infection state, as seen in EBV, the virus is resistant to antiviral drugs and evades immune surveillance, and cancers linked to EBV are often scarcely detectable by the immune system [11]. EBV, the first virus identified as oncogenic, infects approximately 90% of the global population, with primary infection typically acquired via the oral route during childhood or adolescence. This widespread prevalence facilitates the establishment of lifelong latent infection [12].

Moreover, cancers linked to oncoviruses often possess intricate immune-escape mechanisms, such as HBV-induced hepatocellular carcinoma (HCC), which downregulates MHC molecules to evade immune surveillance [13].

Furthermore, oncogenic viruses, such as HBV and HPV, exhibit high variability with multiple subtypes and genetic mutations. Over 200 types of HPV have been identified and described, with at least 14 considered high-risk and potentially leading to cancer development. High-risk types of HPV are associated with various cancers, including cervical, anal, penile, oropharyngeal, vaginal, and vulvar cancers. Two of these types, especially types 16 and 18, are responsible for the majority of HPV-related cancers, accounting for about 70% of cervical cancer cases and precancerous lesions in the cervix [14,15,16]. According to GLOBOCAN, cervical cancer ranks fourth among the most common malignancies in women, both in terms of incidence and mortality. It is estimated that in 2022, there were 660,000 new cases and 350,000 deaths worldwide due to this disease [17]. Notably, HPV is primarily transmitted through sexual activity, but infection can also occur through skin contact. Vaccination against HPV is one of the most effective strategies for preventing HPV-related cancers [14].

Despite the established role of oncogenic viruses in cancer development, awareness of virus–cancer relationships and virus-related cancer prevention, including HPV vaccination, remains insufficient, although existing evidence indicates that higher levels of HPV knowledge are associated with greater vaccine acceptance [18,19,20,21]. Nevertheless, vaccination uptake reflects a complex decision-making process influenced by multiple factors, and its patterns differ across countries and population groups [22,23,24].

Previous studies have identified numerous factors influencing decisions regarding HPV vaccination, most commonly related to limited knowledge, concerns about adverse effects, and a lack of trust in the healthcare system and new vaccines. These factors are referred to in the literature as determinants of vaccine acceptance, understood as influences and contextual conditions shaping vaccination attitudes rather than necessarily reflecting causal relationships [22]. The reluctance or refusal to vaccinate despite the availability of vaccines is defined as “vaccine hesitancy” by the World Health Organization (WHO) and classified as a global health threat [25]. In light of this definition, three distinct behavioral categories related to vaccination can be identified in the literature: vaccine hesitancy, vaccine refusal, and vaccine opposition. Vaccine hesitancy represents a broad spectrum ranging from refusal, through reluctance, such as uncertainty and delay, to partial acceptance (hesitant individuals can accept certain vaccines and still have doubts [22]. Vaccine refusal refers to a deliberate decision not to vaccinate despite vaccine availability, typically driven by specific concerns such as perceived safety risks. In contrast, vaccine opposition denotes a general, unconditional resistance to vaccination, manifesting as a refusal to be vaccinated [26,27,28]. Comparative analyses have shown that factors potentially influencing the decision to vaccinate or refuse vaccination varied depending on the country and population group. Understanding these relationships enables the more effective design of targeted educational interventions. HPV vaccine hesitancy in Europe is a multifactorial phenomenon and requires individualized, locally tailored interventions rather than universal solutions [22].

In response to this gap, the aim of this study was to assess awareness of virus–cancer relationships and willingness to vaccinate against a certain cancer-associated virus among medical and non-medical Polish students. In addition, we evaluated whether HPV-vaccinated students demonstrate greater awareness of the HPV–cancer link compared to unvaccinated students, and examined willingness to vaccinate against a certain cancer-associated virus according to HPV vaccination status. Both in the study aims and throughout the entire study, we refer to a certain cancer-associated virus without specifying its name in order to avoid linking it to any specific virus that contributes to cancer.

## 2. Materials and Methods

### 2.1. Participants, Inclusion and Exclusion Criteria

This study was a cross-sectional survey. The survey included 1013 first- and second-year university students in Poland during the 2023/2024 academic year, recruited using a convenience, non-random sampling approach with voluntary and anonymous participation. The inclusion criteria were as follows: age ≥ 18 years old and current student status. The exclusion criteria included students enrolled in years other than the first or second, as well as students pursuing more than one field of study. Ethical approval was obtained pursuant to Resolution No. 8/2021 of the bioethics commission at the Faculty of Medicine of the Collegium Medicum of the University of Warmia and Mazury in Olsztyn of 24 February 2021.

Participants were divided into two groups. The first group comprised 492 medical students, defined as students enrolled in any health-related programs, including medicine, nursing, emergency medicine, physiotherapy, and dietetics. This definition clarifies that the term “medical student” in this study is not limited to medicine alone. The second group comprised 521 non-medical students, enrolled in fields such as law, administration, informatics, tourism and recreation, and civil engineering. Participants were classified as HPV-vaccinated if they had received at least one dose of the HPV vaccine, including those who had completed the full vaccination schedule and those currently undergoing the vaccination process. Participants who had not received any dose were classified as HPV-unvaccinated. Place of residence was categorized based on population size (rural areas, cities up to 100,000 inhabitants, and cities with more than 100,000 inhabitants). Marital status was self-reported and included four categories: single (not married or in a stable relationship), married or in a stable relationship, divorced, and widowed. A cancer diagnosis referred to any self-reported previous diagnosis of cancer (any type). Family/friends with cancer were defined as a self-reported history of cancer diagnosis in any family member or friend, regardless of cancer type.

### 2.2. Survey Instrument Development, Pilot Testing, and Administration

The purpose-designed survey instrument was developed specifically for this study, following general methodological guidelines [29]. It comprised 10 core closed-ended and 8 sociodemographic items. The English version of the questionnaire is provided in the Supplementary Materials. In the final question of the survey, which assessed respondents’ willingness to vaccinate against a certain cancer-associated virus (i.e., a virus that contributes to cancer), those who were considering refusing vaccination (i.e., selecting either of the “No” options) were instructed to indicate whether their decision was due to a general, unconditional opposition to vaccines or to specific concerns. Respondents who selected “No, because I am opposed to vaccination” were classified as anti-vaccine refusers (unconditional opposition refusers), whereas those who selected “No, other reasons …” were classified as concerns-based refusers and could provide their own reasons, such as concerns about vaccine safety.

A pilot assessment involving 40 individuals was conducted to ensure that all items were clearly understood. Based on the feedback, several questions were refined and adjusted. The revised questionnaire was subsequently subjected to formal validation.

Recruitment was conducted on-site among university students in a classroom during class breaks. The self-administered questionnaire was distributed in paper form by trained interviewers. Eligibility was assessed prior to participation using brief oral screening questions administered by trained interviewers. Individuals younger than 18 years of age and those meeting any of the exclusion criteria outlined in Section 2.2 were excluded from the study. No incentives were offered for participation. Participation was voluntary and anonymous, and completion of the questionnaire constituted informed consent.

### 2.3. Assessment of Comprehensibility, Acceptability, and Reliability

Following pilot refinement, the final version of the questionnaire was evaluated for comprehensibility, acceptability, and reliability. The validation process was conducted in an independent group of 50 Polish university students. A semi-structured interview approach was used to assess comprehensibility and acceptability. Students completed both the main questionnaire and the validation questionnaire. Information regarding the time required to complete the questionnaire, as well as its structure, format, clarity, comprehensibility, and appropriateness, was collected. All respondents indicated that the form of the questionnaire was appropriate, the font size was sufficient, and the questions were understandable. Most respondents reported that the length of the survey was adequate (94%). Only one participant indicated that there were questions they did not wish to answer or found difficult to answer. The average time to complete the survey was 2.1 min (range: 1.0–2.7 min). Respondents also noted that completing the questionnaire made them aware of important aspects they had not recognized before participation in the survey.

Reliability was assessed using a test–retest design with a two-week interval. The repeatability of responses was estimated using Cohen’s Kappa coefficient. The values of the Kappa coefficient range from 0 to 1 and were interpreted as follows: 0.81–1.00, very good repeatability; 0.61–0.80, good repeatability; 0.41–0.60, moderate repeatability; 0.21–0.40, poor repeatability; <0.21, very poor repeatability [30]. The reliability analysis demonstrated very good repeatability for almost all main items, with Cohen’s Kappa values ranging from 0.85 to 1.00. The only exception was the item “Does human papillomavirus (HPV) contribute to oropharyngeal cancer?”, which showed moderate repeatability (Cohen’s Kappa = 0.48).

### 2.4. Statistical Analysis

The chi-square test was used to assess relationships between awareness and factors (sex, place of residence, having cancer patients among family or friends, or type of study). Multivariate analysis was conducted using multiple logistic regression analyses. Specifically, logistic regression models with the backward elimination method were performed to determine the relationships between students’ awareness of virus–cancer links and a set of independent variables (gender, professional status, place of residence, being vaccinated against HPV, and having family or friends diagnosed with cancer). The odds ratio (OR) with a 95% confidence interval (CI) was calculated for the “yes” category of the analyzed questions.

A *p*-value < 0.05 was considered significant. The analysis was conducted using Statistica (data analysis software), version 13 [StatSoft Polska Sp. z o.o., 2024; www.statsoft.pl] (accessed on 6 April 2026).

## 3. Results

### 3.1. Characteristics of the Participants

The study involved 1013 respondents aged 18–23 years, with a mean age of 21 ± 3.5 years. Among them, 288 were men (28.4%), and 713 were women (70.4%). Almost all participants (91.5%) had completed secondary school. The vast majority (62.6%) lived in cities (up to 100,000 inhabitants—29.3% and over 100,000 inhabitants—33.3%), while 37.4% lived in rural areas. Medical students accounted for 48.6% of participants, while non-medical students accounted for 51.4%. Most participants (73.5%) were single (not married or in a stable relationship). Only 1.2% of respondents had been diagnosed with cancer, while over 70% of participants reported having a family member or friend who has ever been diagnosed with cancer (Table 1).

### 3.2. Awareness of Virus–Cancer Links According to Sociodemographic Characteristics

Results regarding respondents’ awareness, based on general questions and detailed questions, are presented in Table 2. Overall, 85.6% of respondents indicated that some viruses are capable of persisting in the human body for many years without causing clinical symptoms. This perception was substantially more common among medical students than among non-medical students (90.0% vs. 81.6%, *p* < 0.001). Similarly, students residing in large cities (with >100,000 inhabitants) were more likely than those from rural areas to acknowledge the possibility of long-term viral latency (90.2% vs. 82.5%, *p* = 0.04). In addition, 71.7% of participants believed that certain viruses may be associated with cancer development. Recognition of this relationship was more frequent among medical students than among non-medical students (75.8% vs. 67.9%, *p* = 0.02).

Focusing on the detailed survey questions, overall awareness of virus–cancer relationships is presented below. Overall, 19.0% of respondents correctly recognized that EBV plays a role in cancer development, while 7.9% answered incorrectly and 73.1% selected “I don’t know”. For the survey questions on virus–cancer awareness, selecting “I don’t know” was considered a lack of awareness. Awareness of the EBV–cancer link was substantially higher among medical students than non-medical students. Only 28.9% of medical students and 9.8% of non-medical students were aware that EBV plays a role in cancer development (*p* < 0.001). Women more often than men indicated that EBV may contribute to cancer development (21.6% vs. 13.2%, *p* = 0.009). Medical students significantly more often than non-medical students (40.5% vs. 26.3%, *p* < 0.001) were aware that viral infections can play a role in the development of lymphoma. Furthermore, respondents with a history of cancer among relatives or friends demonstrated a higher level of awareness compared with those without such exposure (36.3% vs. 24.6%, *p* = 0.002). Overall, 32.0% of respondents were aware that HBV contributes to liver cancer. Awareness was significantly higher among medical students than non-medical students (44.2% vs. 20.5%, *p* < 0.001). Women were significantly more likely than men to know that HBV contributes to liver cancer (35.5% vs. 22.6%, *p* < 0.001). Respondents with relatives or friends affected by cancer also demonstrated higher awareness regarding HBV and liver cancer than those without such a history (33.5% vs. 27.9%, *p* = 0.005). Among all respondents, 43.8% were aware that HPV contributes to cervical cancer. Awareness was significantly higher among respondents from large cities compared with those from rural areas (55.2% vs. 40.5%, *p* < 0.001). Recognition of this relationship was significantly higher among women than men (51.1% vs. 26.0%, *p* < 0.001). Similarly, individuals with relatives or friends diagnosed with cancer were more aware of the HPV–cervical cancer link than those without such experience (48.8% vs. 30.5%, *p* < 0.001). Medical students were substantially more likely than non-medical students to identify HPV as a cause of cervical cancer (62.1% vs. 26.7%, *p* < 0.001).

Overall, 25.4% of respondents were aware that HPV contributes to the development of oropharyngeal cancer, with higher awareness among medical students compared with non-medical students (36.3% vs. 15.2%, *p* < 0.001). Students living in large cities demonstrated higher awareness of this relationship than those from rural areas (32.3% vs. 21.4%, *p* = 0.003). Women more often than men recognized HPV as a risk factor for oropharyngeal cancer (28% vs. 19.8%, *p* = 0.03). Respondents with relatives or friends diagnosed with cancer also showed greater awareness than those without such experience (27.4% vs. 19.8%, *p* = 0.04) (Table 2).

### 3.3. HPV Vaccination Status and Willingness to Vaccinate Against a Cancer-Associated Virus According to Sociodemographic Characteristics

HPV vaccination status and willingness to vaccinate against a cancer-associated virus were analyzed according to selected sociodemographic characteristics (Table 3). Categories with no responses (*n* = 0) were omitted from the tables for clarity. Among the 1013 respondents, 14.5% reported being vaccinated against HPV. Of those not vaccinated, 60.5% had heard of the HPV vaccination, while 24.9% had not heard about it. Significantly more medical than non-medical students were vaccinated against HPV (21.2% vs. 8.2%, *p* < 0.001). Vaccination rates were significantly higher among residents of large cities than among rural areas (19.9% vs. 8.7%, *p* < 0.001), and among women than men (15.9% vs. 11.5%, *p* < 0.001). Participants with relatives or friends who had cancer were also more likely to be vaccinated (15.3% vs. 12.5%, *p* < 0.001) (Table 3).

Regarding willingness to vaccinate against a virus described as contributing to cancer (i.e., a cancer-associated virus), 39.2% of respondents indicated that they would accept vaccination even if the infection risk with this virus were low. An additional 46.6% reported that they would choose vaccination if the infection risk with this virus were relatively high. Eleven point one percent of respondents stated that they would refuse vaccination because they were opposed to vaccination, while 3.0% cited other reasons (Table 3). Among those who refused vaccination for reasons other than an unconditional opposition to vaccination, concerns about vaccine safety (including perceived harms and uncertainty regarding potential adverse effects) were the most common, reported by 53.4% of respondents. Other reasons included the belief of being healthy, young, or strong enough to cope with infection (23.3%), doubts about vaccine effectiveness in preventing infection or severe disease (20.0%), and concerns regarding excessive vaccination, which may overload the immune system (3.3%). Willingness to vaccinate against a cancer-associated virus at low infection risk was significantly higher among medical students (48.3% compared with 30.7% of non-medical students, *p* < 0.001), whereas opposition to vaccination was considerably more frequent among non-medical students (18.2% compared with 3.7%, *p* < 0.001). Participants with relatives or friends affected by cancer were more likely to accept vaccination even at a low perceived risk of infection (42.8% vs. 29.4%, *p* < 0.001), whereas no significant differences were observed according to place of residence or gender (Table 3).

### 3.4. Multivariate Analysis of Factors Associated with Students’ Awareness of Virus–Cancer Links (Sociodemographic Variables and Vaccination Status)

Students vaccinated against HPV were more likely to be aware of HPV’s role in cervical cancer (OR = 8.39, *p* < 0.001) and oropharyngeal cancer (OR = 3.23, *p* < 0.001), compared with the reference group (unvaccinated students who had not heard of HPV vaccination). Similarly, students who were unvaccinated but had heard about HPV vaccination were more likely to be aware of HPV’s role in cervical cancer (OR = 4.71, *p* < 0.001) and oropharyngeal cancer (OR = 3.41, *p* < 0.001), compared with the reference group. They also demonstrated greater awareness that viruses can persist in the human body without causing symptoms (OR = 4.71, *p* < 0.001 and OR = 1.68, *p* = 0.013, respectively).

Medical students were significantly more likely than non-medical students to be aware of HPV’s role in cervical cancer (OR = 2.47, *p* < 0.001) and oropharyngeal cancer (OR = 2.34, *p* < 0.001), as well as to recognize that certain viruses can persist in the body (OR = 1.56, *p* = 0.032) and that some viruses are associated with cancer development (OR = 1.48, *p* = 0.005).

Female participants (OR = 2.08, *p* < 0.001) and students with a family member or friend diagnosed with cancer (OR = 1.74, *p* = 0.001) were more likely to indicate that HPV contributes to cervical cancer (Table 4).

### 3.5. Awareness of the Role of HPV in the Development of Cervical and Oropharyngeal Cancers and Willingness to Vaccinate Against a Cancer-Associated Virus Stratified by HPV Vaccination Status

**Figure 1:**
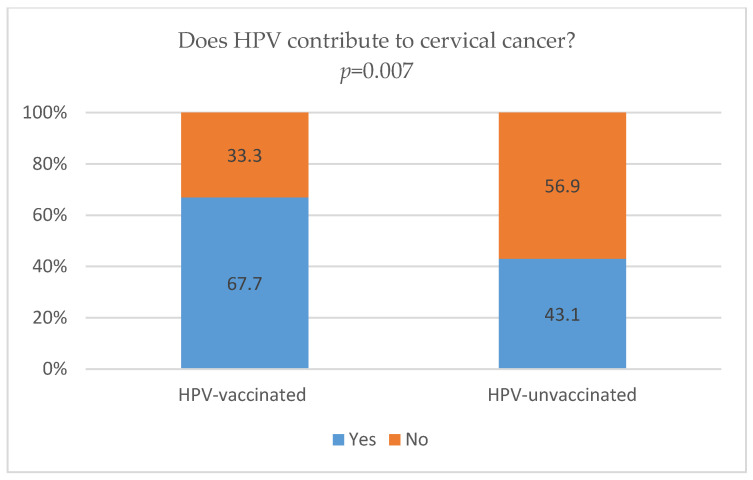
HPV vaccination status and awareness of HPV’s contribution to the development of cervical cancer. Definitions: HPV-vaccinated—participants who had received HPV vaccination; HPV-unvaccinated—participants who had not received HPV vaccination. Abbreviations: HPV—human papillomavirus.

**Figure 2:**
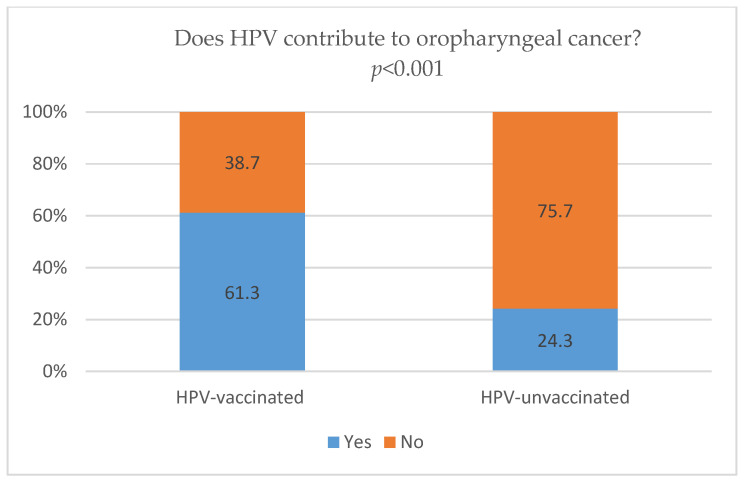
HPV vaccination status and awareness of HPV’s contribution to the development of oropharyngeal cancer. Definitions: HPV-vaccinated—participants who had received HPV vaccination; HPV-unvaccinated—participants who had not received HPV vaccination. Abbreviations: HPV—human papillomavirus.

HPV-vaccinated respondents demonstrated significantly higher awareness of HPV’s contribution to cervical cancer than those in the HPV-unvaccinated group (67.7% vs. 43.1%, *p* = 0.007; Figure 1). Similarly, vaccinated students showed higher awareness that HPV contributes to oropharyngeal cancer compared with unvaccinated peers (61.3% vs. 24.3%, *p* < 0.001; Figure 2).

**Figure 3:**
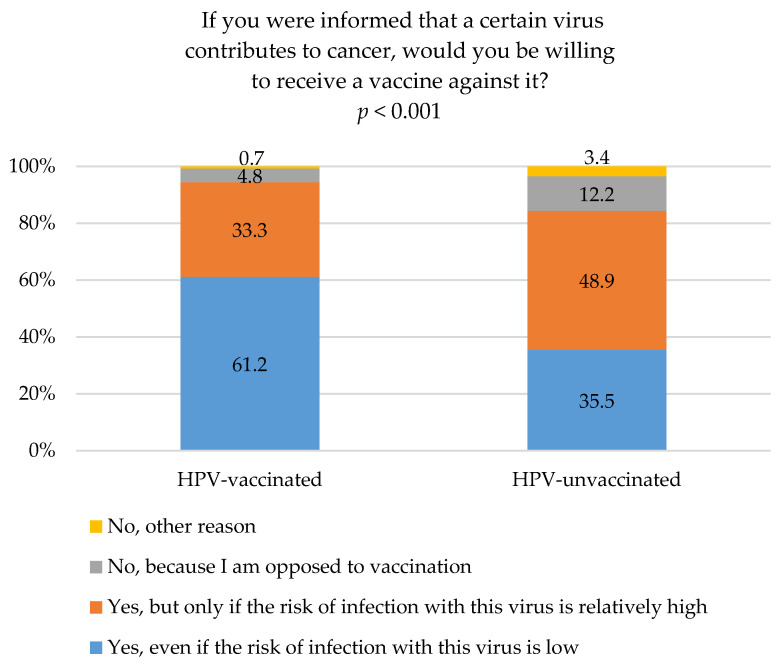
Willingness to vaccinate against a cancer-associated virus stratified by HPV vaccination status. Definitions: HPV-vaccinated—participants who had received HPV vaccination; HPV-unvaccinated—participants who had not received HPV vaccination. Abbreviations: HPV—human papillomavirus.

The willingness to receive a vaccine against a certain cancer-associated virus was expressed by 94.5% of individuals vaccinated against HPV. Among them, 61.2% would get vaccinated even at low perceived infection risk, while 33.3% would do so at high perceived infection risk with that virus. Among individuals vaccinated against HPV, 94.5% expressed willingness to receive a vaccine against a certain cancer-associated virus. Of these, 61.2% would choose vaccination even if the perceived risk of infection were low, whereas 33.3% would do so only if the perceived risk were high. In comparison, 84.4% of HPV-unvaccinated individuals expressed willingness to receive such a vaccine. However, in this group, 35.5% were willing to vaccinate at low perceived infection risk, whereas 48.9% would consider vaccination exclusively at high perceived infection risk from that virus. Significantly more often, those vaccinated against HPV expressed a willingness to be vaccinated against a certain cancer-associated virus, even at low perceived risk of viral infection, compared to HPV-unvaccinated (61.2% vs. 35.5%; *p* < 0.001). It should be emphasized that HPV-unvaccinated students were considerably more likely than HPV-vaccinated individuals to express willingness to be vaccinated against a certain cancer-associated virus when the perceived risk of infection with that virus was high (48.9% vs. 33.3%; *p* < 0.001). Vaccine refusal was observed in both groups; however, HPV-unvaccinated participants were significantly more likely to report unconditional opposition to vaccination compared with HPV-vaccinated individuals (12.2% vs. 4.8%; *p* < 0.001). Refusal based on other concerns was relatively uncommon overall but was also more prevalent among unvaccinated respondents (3.4% vs. 0.7%; *p* < 0.001) (Figure 3).

## 4. Discussion

In line with the study aims, the present findings provide important insights into students’ awareness of virus–cancer relationships and their willingness to vaccinate against a certain cancer-associated virus. The student population is particularly important to study because they represent future parents, whose knowledge and attitudes toward vaccination may influence the health behaviors of the next generation. Understanding their awareness and perceptions may help inform the design of interventions to support vaccine acceptance. These findings may have implications for future public health strategies aimed at improving vaccine uptake and reducing the burden of virus-related cancers.

While the majority of respondents (85.6%) believed that some viruses may persist latently in the human body for many years without causing clinical symptoms, and 71.7% believed that some viruses may be associated with cancer development, their awareness substantially declined when specific virus–cancer associations were assessed. Recognition of individual virus–cancer links remained limited, with awareness rates of 19.0% for EBV, 32.0% for HBV, 43.8% for HPV–cervical cancer, and 25.4% for HPV–oropharyngeal cancer (Table 2).

This discrepancy likely reflects the difference between general specific beliefs and detailed specific awareness of specific associations. Most people believe they understand more than they actually do, due to general overconfidence in their knowledge and skills [31,32]. Contemporary research suggests that this gap reflects limitations in general health literacy and an overreliance on recognition-based reasoning, leading to an overestimation of actual knowledge when assessed using general statements rather than specific, fact-based questions [32,33,34]. Therefore, when participants in our study were asked about specific virus–cancer associations, awareness of the role of EBV in cancer development was limited in both groups of students, reported by 28.9% of medical students and 9.8% of non-medical students.

The observed differences in EBV-related awareness are particularly important in light of evidence that EBV induces tumors by establishing a latent infection in B cells, expressing viral proteins and RNAs that promote proliferation and inhibit apoptosis, contributing to malignancies such as Burkitt’s lymphoma, nasopharyngeal carcinoma, Hodgkin’s lymphoma, and EBV-associated gastric cancer [35,36]. Although associations between EBV and breast cancer and colorectal cancer are still being explored, it is known that factors such as latent EBV infection and inflammation play significant roles in colorectal cancer development [37,38]. Currently, no EBV vaccine has been approved; therefore, prevention relies on general measures, such as avoiding contact with saliva from infected individuals, which can be achieved through public education [39]. Significant educational initiatives are still needed for oncogenic viruses such as EBV.

Awareness of HPV-related cancers deserves particular attention. While HPV is widely recognized as a cause of cervical cancer, our results showed that awareness of both HPV-related cervical and oropharyngeal cancers remains inadequate, especially among non-medical students and male participants (Table 2). In the effort to eliminate cervical cancer by 2030, the Cervical Cancer Elimination Initiative has established national goals of 90-70-90 for countries to achieve. These goals stipulate that 90% of girls should be fully vaccinated with the HPV vaccine by age 15, 70% of women should undergo high-performance screening tests at ages 35 and 45, and 90% of women diagnosed with precancerous lesions or invasive cancer should receive appropriate treatment [40]. Achieving these targets requires not only vaccination and screening but also improving public awareness, which remains uneven across populations.

While some studies [41,42] indicate increasing social awareness of viruses such as HPV and their role in cancer development, the present study suggests that significant educational initiatives are still needed for oncogenic viruses. Among participants in the present survey, medical students were far more likely than non-medical students to recognize HPV as a cause of cervical cancer, with 62.1% of medical students aware of this compared with 26.7% of non-medical students, yet even among medical students, the level of awareness was suboptimal. Only 36.3% of medical students and 15.2% of non-medical students were aware that HPV contributes to the development of oropharyngeal cancer. Similarly, awareness of the role of HBV in liver carcinogenesis was considerably higher among medical students (44.2%) than among non-medical peers (20.5%), although overall awareness was very low (Table 2). The observed differences in HBV-related awareness are particularly important in light of the well-established mechanisms linking chronic hepatitis B infection with hepatocellular carcinoma [13,43]. Our findings regarding gaps in awareness, particularly in the context of HPV-related oropharyngeal cancer, HPV-related cervical cancer, and HBV-related liver cancer, are consistent with other studies [18,20,21,44,45] which demonstrated significant gaps in knowledge about the oncogenic potential of HBV and HPV across different population groups, including healthcare professionals. This underscores the need for targeted educational initiatives across all populations.

Medical students consistently demonstrated higher awareness across all assessed virus–cancer relationships (Table 2), likely reflecting their personal interest, engagement in medical topics. Nevertheless, even among those naturally interested in medicine, awareness of examined relationships—particularly EBV-related cancers and HPV-related oropharyngeal cancer—remained insufficient, highlighting that the issue affects not only non-medical students but society as a whole and requires appropriate educational interventions. While medical education may provide a general foundation for understanding the relationship between oncogenic viruses and cancer, it does not always impart in-depth knowledge, particularly concerning specific malignancies. These findings align with prior research, which showed that although individuals with some medical training demonstrated higher general awareness of environmental carcinogens and cancer risks, a significant portion (including healthcare professionals) lacked clarity regarding specific risk factors, notably the role of viral infections in cancer development [46,47]. This emphasizes the need for improved education and training on oncogenic viruses and their involvement in malignancies to ensure that future healthcare providers are adequately prepared to inform and counsel patients effectively.

Furthermore, in the present study, women demonstrated greater awareness of oncogenic viruses, such as EBV, HBV, and HPV, than men (Table 2). This observed gender-related difference may reflect broader patterns in health-related behaviors, as previous studies have shown that women are more likely to engage in preventive measures and to utilize healthcare services more frequently than men, in turn, facilitating greater exposure to health education and contributing to higher awareness [48,49]. Furthermore, women are more motivated to understand the risks of HPV because the virus poses a direct health threat in the form of cervical cancer, and screening programs, vaccinations, and educational campaigns are primarily targeted at them, increasing their awareness. Recent studies have documented that women generally demonstrate higher awareness and knowledge of HPV and its link to cervical cancer compared to men, likely influenced by targeted cervical cancer screening and vaccination programs [50,51,52]. Consistent with the research mentioned above, the present study demonstrated that more women (51.1%) than men (26.0%) were aware that HPV contributes to cervical cancer (Table 2). This finding was further confirmed in multivariable logistic regression analysis, where female participants had significantly higher odds of recognizing the role of HPV in cervical cancer compared with men (OR = 2.08, *p* < 0.001; Table 4). Women demonstrated higher awareness of HPV as a risk factor for oropharyngeal cancer, with 28% of women recognizing the link compared to 19.8% of men, highlighting a persistent gender disparity in perception. This pattern supports the notion that HPV continues to be perceived primarily as a women’s health issue, despite the growing epidemiological burden of HPV-associated head and neck cancers, particularly among men. This lack of awareness is particularly troubling given the increasing incidence of HPV-related oropharyngeal cancers globally [16]. These findings highlight the need to reframe HPV education as a gender-neutral and population-wide public health concern.

This study highlights the influence of personal cancer experience on awareness. Students who reported having a relative or friend diagnosed with cancer demonstrated significantly greater awareness of HBV-related cancers (Table 2). This finding suggests that emotional or personal exposure to cancer enhances risk perception and motivates health-related learning, likely by making the consequences of infection more tangible. These results reinforce the importance of personalized, experience-based educational approaches in increasing awareness of virus-associated cancers and promoting preventive behaviors. This suggests that personal experience with cancer can significantly impact awareness and health behaviors, a trend supported by previous studies [53,54,55].

Our study demonstrated significant differences not only in awareness but also in willingness to vaccinate against a certain cancer-associated virus between medical and non-medical students. In particular, willingness to vaccinate despite a low risk of infection was significantly higher among medical students (48.3%) than among non-medical students (30.7%), whereas opposition to vaccination was considerably more frequent among non-medical students (18.2%) than among medical students (3.7%) (Table 3). In this context, perceived viral infection risk—understood as an individual’s subjective assessment of the likelihood of harm, the severity of its consequences, and their emotional response to the potential risk [56]—may provide a useful framework for interpreting these differences. In the present study, declared vaccination willingness varied depending on whether the perceived viral infection risk was described as low or relatively high. While 39.2% of respondents declared readiness to vaccinate despite low infection risk, the largest proportion (46.6%) indicated that they would do so if the perceived infection risk was relatively high, highlighting the situational nature of vaccination intentions. Our results suggest that throughout the study population, higher perceived risk of infection is associated with greater willingness to vaccinate, a pattern also observed in other studies and consistent with the Health Belief Model [57,58]. Among those who refused vaccination, the majority (11.1%) did so because they were unconditionally opposed to vaccination, and only 3% of participants cited other reasons, such as safety concerns, perceived personal health, or doubts about vaccine effectiveness. Respondents with relatives or friends affected by cancer were also more likely to accept vaccination despite low perceived infection risk, suggesting that personal experience with cancer strongly influences attitudes toward vaccination against a cancer-associated virus (Table 3). These findings are consistent with previous research showing that vaccine refusal is often driven by general opposition or mistrust, while situational factors such as perceived risk, information gaps, and personal experience can strongly influence vaccination intentions [22,59,60,61].

To further contextualize these findings, we also assessed actual HPV vaccination uptake among our respondents. This allowed us to evaluate whether students who had received the HPV vaccine demonstrate greater awareness of cancer-related HPV compared to unvaccinated students, and to examine their willingness to vaccinate against a certain cancer-associated virus according to HPV vaccination status. Our study demonstrated significant differences in HPV vaccination between medical and non-medical students. Medical students showed significantly higher HPV vaccination uptake (21.2% compared with 8.2% among non-medical students) (Table 3), reflecting greater vaccine acceptance in this group. A particularly concerning finding from our study was that a substantial proportion of non-medical students (41.3%) had never heard of HPV vaccination, while a small but noteworthy proportion of medical students (7.5%) were also unaware of it (Table 3). Similar trends have been reported internationally, with non-medical students and the general population consistently showing lower vaccination knowledge than individuals with medical education [62,63,64]. In Poland, a nationwide HPV vaccination program was launched on June 1, 2023, as part of the National Oncology Strategy 2020–2030 [19]. The program provides free vaccination for girls and boys, with vaccines that include 2-valent and 9-valent formulations [65,66]. Despite these efforts, early program data indicate low vaccination coverage (9.8%) and a marked gender imbalance (65% girls compared with 35% boys) [67]. Poland remains among the last European countries to implement a universal HPV vaccination program [68], which is particularly concerning given that cervical cancer ranks sixth among female cancers in Poland compared with tenth in Europe [69,70]. In light of these results, targeted educational interventions should be implemented, particularly for non-medical students and men. Information campaigns aimed at young adults should be developed to improve vaccine coverage and reduce the burden of HPV-related cancers in Poland.

Awareness of the link between HPV and the development of cancer differed significantly between participants based on their vaccination status. Our findings indicate that HPV-vaccinated students have higher awareness of the contribution of HPV to the development of both cervical and oropharyngeal cancers than their unvaccinated peers. Although 67.7% of vaccinated students were aware of HPV’s contribution to cervical cancer, awareness among unvaccinated participants remained substantially lower (43.1%). A comparable disparity was observed for oropharyngeal cancer, where 61.3% of vaccinated students demonstrated awareness of HPV’s contribution, compared with only 24.3% of those who were unvaccinated (Figure 1 and Figure 2). It should also be noted that the test–retest reliability of this item was moderate (κ = 0.48), suggesting greater response variability and possible uncertainty in interpretation of this association. These results are consistent with previous studies indicating that individuals with greater knowledge are more likely to accept vaccination [18,20]. Our study did not investigate whether vaccination precedes knowledge gains or whether better-informed individuals are more likely to get vaccinated. Because the study was cross-sectional, no causal relationship can be inferred from our data. Furthermore, a meta-analysis [71] suggests that substantial knowledge gaps regarding HPV and its oncogenic potential persist among university students, indicating that undergoing vaccination alone may not necessarily be a proxy for health literacy. In line with these observations, our findings indicate that students who have been vaccinated against HPV, compared with those who have not been vaccinated, were more likely to be aware of HPV’s role in cervical cancer (OR = 8.39, *p* < 0.001) and oropharyngeal cancer (OR = 3.23, *p* < 0.001) (Table 4). However, despite this association, a considerable proportion of vaccinated students lacked awareness of the relationships between HPV and these cancers, with over 33% failing to correctly identify the link with cervical cancer and nearly 39% failing to recognize the link with oropharyngeal cancer (Figure 1). Similar gaps were documented by de Oliveira et al. [18], who noted that despite the positive correlation between vaccination and knowledge, approximately 36% of their study population still failed to recognize HPV as a cause of cervical cancer. These findings suggest that being HPV-vaccinated does not necessarily mean that a person has full awareness of the link between HPV and cancer. In this context, education regarding HPV-related cancers may also be considered among vaccinated individuals. Such an approach may be relevant in light of persistent gaps in knowledge observed across different groups, including both vaccinated and unvaccinated students. This may be particularly relevant among young adults, who represent a key target population for HPV-related health education [23,71].

In addition to awareness of the role of HPV in cervical and oropharyngeal cancers, we examined willingness to vaccinate against a cancer-associated virus, with both measures stratified by HPV vaccination status. HPV vaccination status should not be interpreted causally with respect to willingness to receive other vaccines. In this context, HPV vaccination status may serve as a proxy indicator of pre-existing vaccination attitudes and health-related behaviors, thereby reflecting underlying differences between groups rather than an independent effect of vaccination. HPV-vaccinated students were significantly more willing to vaccinate against a cancer-associated virus, particularly at low perceived infection risk, compared with unvaccinated students (61.2% vs. 35.5%; Figure 3). This pattern may partly reflect the well-documented tendency for individuals with prior positive vaccination experience to seek future vaccinations, indicating a more preventive health orientation among HPV-vaccinated students even at low perceived infection risk [72].

In contrast, HPV-unvaccinated students expressed willingness to vaccinate against a cancer-associated virus mainly under high perceived infection risk (48.9% vs. 33.3%), indicating that their willingness increases with higher perceived risk and emphasizing the role of perceived risk in motivating vaccination decisions (Figure 3). These patterns indicate that individuals’ perceptions of susceptibility and severity influence health-related decisions, including vaccination. This suggests that willingness among unvaccinated students may increase in the context of a high perceived risk of infection, highlighting that addressing perceived risk could be crucial for improving vaccine uptake among this group. This interpretation is supported by evidence showing that risk perceptions—including perceived likelihood, susceptibility, and severity—significantly predict vaccination behavior, with consistent effect sizes across studies [57,58,73]. These findings reinforce that perceived infection risk plays a meaningful role in shaping vaccination decisions, aligning with the patterns observed in our sample. These findings are also consistent with research showing that lower perceived risk of HPV related cancers is associated with lower vaccine uptake. For example, parents who did not vaccinate their children against HPV reported lower perceived risk and higher confidence in preventing infection without vaccination [74]. Likewise, enhancing knowledge, building trust, and providing a realistic perception of HPV-related risk have been identified as key strategies to reduce HPV vaccine hesitancy [75]. Together, these studies support the notion that interventions targeting perceived infection risk could help improve vaccination rates among hesitant or unvaccinated populations.

Vaccine refusal was observed in both groups; however, HPV-unvaccinated participants were significantly more likely to report unconditional opposition to vaccination compared with vaccinated participants (12.2% vs. 4.8%, Figure 3). These findings may indicate that low vaccination uptake against cancer-associated viruses could be more related to low perceived risk than to inherently negative attitudes toward vaccination. A small proportion of HPV-vaccinated participants (4.8%) reported unconditional opposition (Figure 3). This finding suggests heterogeneity in attitudes even within vaccinated individuals. It highlights the importance of educational approaches addressing vaccine-related perceptions across different subgroups. Similarly, even among individuals aware of anti-vaccination arguments, a substantial proportion still chose to vaccinate, reinforcing the need for targeted educational interventions even among HPV-vaccinated [76].

These findings suggest a more proactive or preventive-oriented health behavior among HPV-vaccinated individuals, although willingness to vaccinate may be influenced by perceived infection risk. Importantly, these findings have direct practical implications for public health. Health education and vaccination programs should clearly communicate infection risk, as a higher perceived risk is associated with increased willingness to vaccinate. Providing information on the magnitude of cancer-associated viral infection risk may be particularly important for individuals who would act only if infection risk is high. Therefore, future initiatives should emphasize the link between HPV and cancer while explicitly addressing perceived infection risk. In the context of cancer prevention, perception of viral infection risk should be considered a key element in the design of future health education and vaccination initiatives and remain a subject of ongoing scientific investigation as part of strategies aimed at increasing vaccine uptake.

This study has several limitations that should be considered when interpreting the findings. First, its cross-sectional design precludes any causal inferences between awareness, vaccination status, and willingness to vaccinate. The observed associations may reflect underlying characteristics, such as pre-existing health orientation or interest in medical topics, rather than direct effects of educational background. Because temporality and directionality cannot be established, unmeasured confounding is also possible, and the association between vaccination and greater awareness should be interpreted cautiously. Prospective cohort or interventional studies would be needed to determine whether vaccination precedes knowledge gains or whether better-informed individuals are more likely to seek vaccination. Second, the study relied on self-reported data, which may be subject to recall bias and social desirability bias, particularly regarding vaccination status and attitudes toward preventive behaviors. The reproducibility of the question regarding HPV and oropharyngeal cancer was moderate (κ = 0.48), indicating limited test–retest stability of responses for this specific item. We also did not collect detailed information on the exact age at vaccination or the specific motivations for receiving the HPV vaccine, which might have helped explain the variation in attitudes observed even among vaccinated participants. Moreover, we did not assess psychological constructs such as self-efficacy in preventing infection without vaccination or response efficacy related to the perceived effectiveness of the vaccine itself, which may influence vaccination intentions. Third, the sample consisted exclusively of students, which limits the generalizability of the results to the broader population, especially to older age groups or individuals with different educational backgrounds. Moreover, the sample was restricted to first- and second-year students and was characterized by a substantial gender imbalance with a predominance of women, which may further affect its representativeness. Finally, willingness to vaccinate was assessed using a hypothetical scenario based on perceived infection risk, which may not fully reflect real-world vaccination behavior. By design, this study used an abstract term, “a certain cancer-associated virus,” to minimize potential bias related to pre-existing perceptions or attitudes toward specific viruses and their vaccines. However, this approach may limit the clinical interpretability and generalizability of the findings, as vaccination attitudes can vary depending on the specific pathogen and available vaccine. Therefore, the findings should be interpreted with caution, and further longitudinal and population-based studies are warranted to confirm and extend these observations.

## 5. Conclusions

Awareness of virus–cancer links among Polish students remains insufficient. Although medical students demonstrated higher awareness than their non-medical peers, overall awareness in both groups is inadequate. Awareness of HPV-related cervical cancers was particularly low among men, non-medical students, and individuals without a family or friend history of cancer. These findings highlight the influence of gender and underscore the need to reframe HPV education as a gender-neutral, population-wide public health concern, while emphasizing targeted interventions for men. HPV vaccination coverage in the study population was low, and a substantial proportion of both medical and non-medical students had never heard of the HPV vaccine. This reveals critical gaps in basic awareness that HPV vaccination exists and underscores the need for focused educational efforts to improve vaccine uptake and reduce the burden of HPV-related cancers. Although HPV-vaccinated students showed greater awareness of HPV’s role in cervical and oropharyngeal cancers than unvaccinated peers, a substantial awareness gap persists, particularly among the unvaccinated. Willingness to vaccinate against a certain cancer-associated virus varied according to perceived infection risk. HPV-unvaccinated students expressed willingness to vaccinate against a certain cancer-associated virus, mainly under high perceived infection risk compared with vaccinated students, suggesting that high perceived risk may be a key driver of vaccine willingness among unvaccinated individuals.

Overall, these results may suggest a need for targeted educational interventions to improve both awareness of virus–cancer links and risk perception, potentially supporting greater acceptance of cancer-associated virus vaccinations. They also highlight the importance of ongoing education for both HPV-vaccinated and unvaccinated individuals to ensure informed, evidence-based health decisions.

## Figures and Tables

**Table 1:** Sociodemographic (including health-related) characteristics of the study population.

Variable	Categories	*n* = 1013	%
Gender	Female	713	70.4
	Male	288	28.4
	Prefer not to answer this question	9	0.9
	No data	3	0.3
Level of education	Primary education	3	0.3
	Secondary education	927	91.5
	Higher education—bachelor’s degree	73	7.2
	Higher education—master’s degree	10	1.0
Place of residence	Rural areas	379	37.4
	City ≤ 100,000	297	29.3
	City > 100,000	337	33.3
Professional status	Medical student	492	48.6
	Non-medical student	521	51.4
Marital status	Married or in a stable relationship	263	26.0
	Single	745	73.5
	Divorced	4	0.4
	Widowed	1	0.1
Participant diagnosed with cancer	Yes	12	1.2
	No	1000	98.7
	No data	1	0.1
Family or friends with cancer	Yes	740	73.0
	No	272	26.9
	No data	1	0.1

Definitions: City ≤ 100,000—city with up to 100,000 inhabitants; City > 100,000—city with more than 100,000 inhabitants.

**Table 2:** Relationships between sociodemographic characteristics and awareness of virus–cancer links among Polish students.

Survey Question	Answer Category	All		Medical		Non-Medical			Rural Areas		City ≤ 100,000		City > 100,000			Woman		Men			Family/Friends with Cancer		Family/Friends Without Cancer		
		*n* = 1013	%	*n*	%	*n*	%	*p*	*n*	*%*	*n*	%	*n*	%	*p*	*n*	%	*n*	%	*p*	*n*	%	*n*	%	*p*
Do you think that there are any viruses that can persist in the human body for many years without causing disease symptoms?	Yes	867	85.6	442	90	425	81.6	<0.001	312	82.5	251	84.5	304	90.2	0.04	616	86.5	241	83.7	0.48	642	86.8	225	82.7	0.25
	No	58	5.7	16	3.3	42	8		24	6.4	18	6.1	16	4.8		38	5.3	20	6.9		40	5.4	18	6.6	
	I don’t know	87	8.6	33	6.7	54	10.4		42	11.1	28	9.4	17	5		58	8.2	27	9.4		58	7.8	29	10.7	
	No data	1	0.1																						
Do you think that there are any viruses that may be associated with cancer development?	Yes	726	71.7	372	75.8	354	67.9	0.02	273	72.2	205	69	248	73.6	0.52	514	72.2	200	69.4	0.55	537	72.6	189	69.5	0.43
	No	93	9.2	37	7.5	56	10.7		30	8	30	10.1	33	9.8		62	8.7	31	10.8		63	8.5	30	11	
	I don’t know	193	19.0	82	16.7	111	21.3		75	19.8	62	20.9	56	16.6		136	19.1	57	19.8		140	18.9	53	19.5	
	No data	1	0.1																						
Can the Epstein–Barr virus (EBV), which causes mononucleosis, contribute to cancer development?	Yes	193	19.0	142	28.9	51	9.8	<0.001	71	18.8	46	15.5	76	22.6	0.24	154	21.6	38	13.2	0.009	151	20.4	42	15.4	0.13
	No	80	7.9	25	5.1	55	10.6		31	8.2	26	8.7	23	6.8		53	7.4	24	8.3		54	7.3	26	9.6	
	I don’t know	739	73.1	324	66	415	79.7		276	73	225	75.8	238	70.6		505	71	226	78.5		535	72.3	204	75	
	No data	1	0.1																						
Is there a virus that can contribute to the development of lymphoma?	Yes	336	33.2	199	40.5	137	26.3	<0.001	123	32.5	91	30.6	122	36.2	0.65	247	34.7	83	28.8	0.09	269	36.3	67	24.6	0.002
	No	74	7.3	30	6.1	44	8.4		27	7.2	24	8.1	23	6.8		46	6.5	27	9.4		51	6.9	23	8.5	
	I don’t know	602	59.4	262	53.4	340	65.3		228	60.3	182	61.3	192	57		419	58.8	178	61.8		420	56.8	182	66.9	
	No data	1	0.1																						
Is there a virus that can contribute to the development of leukemia?	Yes	369	36.4	189	38.5	180	34.5	0.10	143	37.8	113	38	113	33.5	0.60	268	37.6	95	33	0.38	277	37.4	92	33.8	0.10
	No	153	15.01	81	16.5	72	13.8		51	13.5	46	15.5	56	16.6		104	14.6	45	15.6		119	16.1	34	12.5	
	I don’t know	490	48.4	221	45	269	51.6		184	48.7	138	46.5	168	49.9		340	47.8	148	51.4		344	46.5	146	53.7	
	No data																								
Does hepatitis B virus (HBV) contribute to liver cancer?	Yes	324	32.0	217	44.2	107	20.5	<0.001	122	32.3	80	26.9	122	36.2	0.17	253	35.5	65	22.6	<0.001	248	33.5	76	27.9	0.005
	No	43	4.2	16	3.3	27	5.2		17	4.5	14	4.7	12	3.6		26.0	3.7	17	5.9		23	3.1	20	7.4	
	I don’t know	645	63.7	258	52.5	387	74.3		239	63.2	203	68.4	203	60.2		433	60.8	206	71.5		469	63.4	176	64.7	
	No data	1	0.1																						
Does human papillomavirus (HPV) contribute to cervical cancer?	Yes	444	43.8	305	62.1	139	26.7	<0.001	153	40.5	105	35.3	186	55.2	<0.001	364	51.1	75	26	<0.001	361	48.8	83	30.5	<0.001
	No	40	4	6	1.2	34	6.5		13	3,4	16	5.4	11	3.3		18	2.5	22	7.6		24	3.2	16	5.9	
	I don’t know	528	52.1	180	36.7	348	66.8		212	56.1	176	59.3	140	41.5		330	46.4	191	66.4		355	48	173	63.6	
	No data	1	0.1																						
Does human papillomavirus (HPV) contribute to oropharyngeal cancer?	Yes	257	25.4	178	36.3	79	15.2	<0.001	81	21.4	67	22.6	109	32.3	0.003	199	28	57	19.8	0.03	203	27.4	54	19.8	0.04
	No	96	9.5	40	8.1	56	10.7		30	8	34	11.4	32	9.5		67	9.4	28	9.7		70	9.5	26	9.6	
	I don’t know	659	65	273	55.6	386	74.1		267	70.6	196	66	196	58.2		446	62.6	203	70.5		467	63.1	192	70.6	
	No data	1	0.1																						

Definitions: City ≤ 100,000—city with up to 100,000 inhabitants; City > 100,000—city with more than 100,000 inhabitants.

**Table 3:** Relationships between sociodemographic characteristics, HPV vaccination status, and willingness to vaccinate against a cancer-associated virus.

Survey Question	Answer Category	All		Medical		Non-Medical			Rural Areas		City ≤ 100,000		City > 100,000			Woman		Men			Family/Friends with Cancer		Family/Friends Without Cancer		
		*n* = 1013	%	*n*	%	*n*	%	*p*	*n*	%	*n*	%	*n*	%	*p*	*n*	%	*n*	%	*p*	*n*	%	*n*	%	*p*
Have you been vaccinated against human papillomavirus (HPV)?	Yes, I have completed the full vaccination schedule	147	14.5	104	21.2	43	8.2	<0.001	33	8.7	47	15.8	67	19.9	<0.001	113	15.9	33	11.5	<0.001	113	15.3	34	12.5	<0.001
	No, but I have heard about this vaccination	613	60.5	350	71.3	263	50.5		222	58.7	179	60.3	212	62.9		465	65.3	142	49.3		466	63.0	147	54.0	
	No, and I have not heard about this vaccination	252	24.9	37	7.5	215	41.3		123	32.6	71	23.9	58	17.2		134	18.8	113	39.2		161	21.7	91	33.5	
	No data	1	0.1																						
If you were informed that a certain virus contributes to cancer, would you be willing to receive a vaccine against it?	Yes, even if the risk of infection with this virus is low	397	39.2	237	48.3	160	30.7	<0.001	142	37.5	117	39.4	138	40.9	0.82	289	40.6	104	36.1	0.08	317	42.8	80	29.4	<0.001
	Yes, but only if the risk of infection with this virus is relatively high	472	46.6	225	45.8	247	47.4		184	48.7	134	45.1	154	45.7		334	46.9	133	46.2		328	44.3	144	52.9	
	No, because I am opposed to vaccination	113	11.1	18	3.7	95	18.2		43	11.4	37	12.5	33	9.8		74	10.4	38	13.2		75	10.2	38	14.0	
	No, other reasons	30	3.0	11	2.2	19	3.7		9	2.4	9	3.0	12	3.6		15	2.1	13	4.5		20	2.7	10	3.7	
	No data	1	0.1																						

Note: Only response categories selected by respondents are presented (categories with *n* = 0, i.e., not selected by any respondents, were not included in the table). Definitions: City ≤ 100,000—city with up to 100,000 inhabitants; City > 100,000—city with more than 100,000 inhabitants.

**Table 4:** Multiple logistic regression models to explain the relationship between the students’ awareness of virus–cancer links and a set of predictor variables.

Effect	Level of Effect	OR (95% CI)	*p*-Value
		Do you think that there are any viruses that can persist in the human body for many years without causing disease symptoms?	
Have you been vaccinated against human papillomavirus (HPV)?	Yes, I have completed the full vaccination schedule	4.71 (2.03–10.92)	<0.001
	No, but I have heard about this vaccination	1.68 (1.12–2.53)	0.013
	No, and I have not heard about this vaccination	1.00	
Professional status	Medical student	1.56 (1.04–2.34)	0.032
	Non-medical student	1.00	
		Do you think that there are any viruses that may be associated with cancer development?	
Professional status	Medical student	1.48 (1.13–1.96)	0.005
	Non-medical student	1.00	
		Does human papillomavirus (HPV) contribute to cervical cancer?	
Have you been vaccinated against human papillomavirus (HPV)?	Yes, I have completed the full vaccination schedule	8.39 (4.82–14.60)	<0.001
	No, but I have heard about this vaccination	4.71 (3.03–7.30)	<0.001
	No, and I have not heard about this vaccination	1.00	
Professional status	Medical student	2.47 (1.83–3.32)	<0.001
	Non-medical student	1.00	
Gender	Female	2.08 (1.47–2.92)	<0.001
	Male	1.00	
Place of residence	Rural areas	1.00	
	City ≤ 100,000	0.68 (0.47–0.97)	0.034
	City > 100,000	1.40 (0.99–1.97)	0.058
Family or friends with cancer	Yes	1.74 (1.24–2.43)	0.001
	No	1.00	
		Does human papillomavirus (HPV) contribute to oropharyngeal cancer?	
Have you been vaccinated against human papillomavirus (HPV)?	Yes, I have completed the full vaccination schedule	3.23 (1.78–5.86)	<0.001
	No, but I have heard about this vaccination	3.41 (2.07–5.61)	<0.001
	No, and I have not heard about this vaccination	1.00	
Professional status	Medical student	2.34 (1.70–3.22)	<0.001
	Non-medical student	1.00	

Definitions: City ≤ 100,000—city with up to 100,000 inhabitants; City > 100,000—city with more than 100,000 inhabitants. Abbreviations: OR—odds ratio; CI—confidence interval.

## Data Availability

All data are presented in this study, and details may be made available upon request from the corresponding author. The data consist of anonymous responses from respondents’ surveys and contain no personally identifiable information.

## Supplementary Materials

The following supporting information can be downloaded at: https://www.mdpi.com/article/10.3390/vaccines14040335/s1, Questionnaire—English version.
