# Supplementary material for: Awareness of Virus–Cancer Links and Willingness to Vaccinate Against a Cancer-Associated Virus by HPV Vaccination Status Among Polish Students: A Cross-Sectional Study
Source: Vaccines (Basel). 2026 Apr 9;14(4):335. doi: 10.3390/vaccines14040335 (PMC13120187; doi:10.3390/vaccines14040335)
Supplement: Supplementary file 1 [file vaccines-14-00335-s001.zip › vaccines-4216788-supplementary.pdf]

Questionnaire

Core Questions

1. Do you think that there are any viruses that can persist in the human body for many years without causing disease symptoms?

   - ☐ Yes
   - ☐ No
   - ☐ I don’t know

2. Do you think that there are any viruses that may be associated with cancer development?

   - ☐ Yes
   - ☐ No
   - ☐ I don’t know

3. Can the Epstein-Barr virus (EBV), which causes mononucleosis, contribute to cancer development?

   - ☐ Yes
   - ☐ No
   - ☐ I don’t know

4. Is there a virus that can contribute to the development of lymphoma?

   - ☐ Yes
   - ☐ No
   - ☐ I don’t know

5. Is there a virus that can contribute to the development of leukemia?

   - ☐ Yes
   - ☐ No
   - ☐ I don’t know

6. Does hepatitis B virus (HBV) contribute to liver cancer?

   - ☐ Yes
   - ☐ No
   - ☐ I don’t know

7. Does human papillomavirus (HPV) contribute to cervical cancer?

   - ☐ Yes
   - ☐ No
   - ☐ I don’t know

8. Does human papillomavirus (HPV) contribute to oropharyngeal cancer?
   - ☐ Yes
   - ☐ No
   - ☐ I don't know

9. Have you been vaccinated against human papillomavirus (HPV)?
   - ☐ Yes, I have completed the full vaccination schedule
   - ☐ Yes, I am currently undergoing vaccination (I have received at least one dose)
   - ☐ No, but I have heard about this vaccination
   - ☐ No, and I have not heard about this vaccination
   - ☐ I don't know, but I have heard about this vaccination
   - ☐ I don't know, and I have not heard about this vaccination

10. If you were informed that a certain virus contributes to cancer, would you be willing to receive a vaccine against it?
    - ☐ Yes, even if the risk of infection with this virus is low
    - ☐ Yes, but only if the risk of infection with this virus is relatively high
    - ☐ No, because I am opposed to vaccination
    - ☐ No, because ………………………………
    - ☐ I don't know
    - ☐ I do not wish to answer this question

Socio-demographic Questions

1. Please indicate your age: … years

2. Please indicate your gender:
   - ☐ Female
   - ☐ Male
   - ☐ Prefer not to answer this question

3. Please indicate your level of education:
   - ☐ Primary education
   - ☐ Secondary education
   - ☐ Higher education – bachelor's degree
   - ☐ Higher education – master's degree

4. Please indicate your place of residence:
   - ☐ Rural area

- ☐ City with up to 100,000 inhabitants
- ☐ City with more than 100,000 inhabitants

5. Please indicate your professional status:
   - ☐ Student of a medical study; field of study…………………..; year of study...…
   - ☐ Student of a non-medical study; field of study………………..; year of study...…

6. Please indicate your marital status:
   - ☐ Married or in a stable relationship
   - ☐ Single
   - ☐ Divorced
   - ☐ Widowed

7. Have you ever been diagnosed with cancer?
   - ☐ Yes
   - ☐ No

8. Have any of your family or friends ever been diagnosed with cancer?
   - ☐ Yes
   - ☐ No
